# Use of sucrose to diminish pore formation in freeze-dried heart valves

**DOI:** 10.1038/s41598-018-31388-4

**Published:** 2018-08-28

**Authors:** Andrés Vásquez-Rivera, Harriëtte Oldenhof, Daniele Dipresa, Tobias Goecke, Artemis Kouvaka, Fabian Will, Axel Haverich, Sotirios Korossis, Andres Hilfiker, Willem F. Wolkers

**Affiliations:** 1 Institute of Multiphase Processes, Leibniz Universität Hannover, Hannover, Germany; 2 Unit for Reproductive Medicine, Clinic for Horses, University of Veterinary Medicine Hannover, Hannover, Germany; 3 Department of Cardiothoracic, Transplantation and Vascular Surgery, Hannover Medical School, Hannover, Germany; 4 Leibniz Research Laboratories for Biotechnology and Artificial Organs, Hannover Medical School, Hannover, Germany; 5 Lower Saxony Centre for Biomedical Engineering Implant Research and Development, Hannover Medical School, Hannover, Germany; 6 LLS ROWIAK LaserLabSolutions, Hannover, Germany

## Abstract

Freeze-dried storage of decellularized heart valves provides easy storage and transport for clinical use. Freeze-drying without protectants, however, results in a disrupted histoarchitecture after rehydration. In this study, heart valves were incubated in solutions of various sucrose concentrations and subsequently freeze-dried. Porosity of rehydrated valves was determined from histological images. In the absence of sucrose, freeze-dried valves were shown to have pores after rehydration in the cusp, artery and muscle sections. Use of sucrose reduced pore formation in a dose-dependent manner, and pretreatment of the valves in a 40% (w/v) sucrose solution prior to freeze-drying was found to be sufficient to completely diminish pore formation. The presence of pores in freeze-dried valves was found to coincide with altered biomechanical characteristics, whereas biomechanical parameters of valves freeze-dried with enough sucrose were not significantly different from those of valves not exposed to freeze-drying. Multiphoton imaging, Fourier transform infrared spectroscopy, and differential scanning calorimetry studies revealed that matrix proteins (i.e. collagen and elastin) were not affected by freeze-drying.

## Introduction

Heart valve diseases are one of the main causes of death worldwide and represent an increasing problem in medical practice^1^. Currently, heart valve dysfunction is typically treated via replacement with mechanical or biological prostheses. Risks associated with mechanical valves include hemorrhage and thromboembolism^2^. Bioprosthetic valves are made by treating bovine or porcine tissue with glutaraldehyde. These glutaraldehyde-treated tissue matrices are inherently different from native matrices because proteins are irreversibly cross-linked^3^. Bioprosthetic valves have been implicated in fibrosis, calcification, degeneration and immunogenic complications^4^. Decellularized heart valves hold promise as alternative for mechanical or bioprosthetic valves. The histoarchitecture of decellularized heart valves closely resembles that of native tissue, and implanted valves have been shown to be repopulated with autologous cells resulting in tissue regeneration and growth^5^. The use of decellularized heart valves to replace a malfunctioning heart valve reduces the risk of immunogenic rejection, whereas anti-coagulation therapy is generally not required^6^. In the surgical practice, sufficient availability of decellularized heart valves of different sizes is required. To satisfy this demand, appropriate preservation methods are needed to preserve valves until they are required. If it were possible to dry heart valves without damaging the tissue, this would increase their shelf-life and ensure off-the-shelf availability, while decreasing the incidence of bacterial contamination. Freeze-drying allows for easy transport and room temperature storage.

Biological materials are generally prone to degradation, particularly when they are immersed in water and at higher temperatures. Previous studies have shown that heart valves can be stored at 4 °C in 50‒80% sucrose solutions or in pure glycerol for up to 52 and 12 weeks, respectively^6,7^. However, stored samples showed separation and clumping of collagen as well as changes in microscopic structure and elastic properties. Moreover, valves that are stored in liquid have an increased risk of bacterial contamination during prolonged storage. These storage problems can be addressed by decreasing the storage temperature and/or drying the sample, which can be achieved by cryopreservation or freeze-drying approaches.

The majority of homograft valves are cryopreserved using 10% dimethyl sulfoxide as cryoprotective agent, and slow cooling at a rate of 1 °C min^−1^ down to −150 °C, followed by storage in the vapor phase of liquid nitrogen^8^. This cryopreservation procedure, however, has been shown to cause alterations in the extracellular matrix caused by ice formation in the tissue^8,9^. Vitrification, or ice-free cryopreservation, results in less damage to the histoarchitecture and tissue proteins compared to normal cryopreservation procedures, and results in less inflammation after transplantation^10^. Both cryopreservation and vitrification require relatively expensive devices for low temperature storage and transport, which are not required for freeze-dried samples.

Whereas freeze-drying is widely used to dry biological materials used in pharmaceutical and food sciences, it is not frequently used to preserve mammalian cells or (decellularized) tissues^11–13^. The efficacy of freeze-dried homografts has been investigated in the 1960s and 1970s, but tests on *in vivo* performance yielded unsatisfactory results^14–16^. Curtil *et al*.^17^ reported that freeze-drying of allograft heart valves resulted in porous leaflets after rehydration. Scaffold porosity (i.e. pore size and distribution) has been shown to be affected by the freeze-drying conditions^18–20^. No lyoprotectants were used in the above mentioned studies to protect the tissues from the damaging effects of freeze-drying.

We have pioneered in freeze-drying of decellularized heart valves using sucrose as lyoprotectant^21^. Incubation of heart valves in sucrose solutions prior to freeze-drying, reduces the adverse effects of freeze-drying by reducing ice crystal formation and by forming a protective glassy state^22^. Sucrose reduces pore formation in freeze-dried tissue and thereby preserves the extracellular matrix (ECM) histoarchitecture. However, pre-treatment with very high sucrose concentrations was shown to be needed (80% w/v) to avoid pore formation^22^. We have recently demonstrated that, if used for transplantation, sucrose-protected freeze-dried decellularized pulmonary heart valves show excellent clinical performance and cell repopulation potential in juvenile sheep^23^.

In this study, the effect of the sucrose concentration used for freeze-drying of heart valves was studied in detail. One of the aims was to investigate the minimal sucrose concentration that is required to diminish pore formation in freeze-dried heart valves. Decellularized heart valves were freeze-dried using sucrose at concentrations ranging from 0 to 80% (w/v), and pore formation was quantified from histological image analysis. Fourier transform infrared spectroscopy (FTIR) was used to evaluate the overall protein secondary structure in freeze-dried heart valves, and differential scanning calorimetry (DSC) was used to determine characteristic protein denaturation profiles. In order to assess intactness and appearance of collagen and elastin in the valves multiphoton imaging was used. In addition, biomechanical properties of freeze-dried heart valves were evaluated under low-strain-rate uniaxial tensile loading to failure.

In the absence of sucrose, freeze-dried valves were shown to have pores after rehydration in the cusp, artery and muscle sections. Use of sucrose was shown to reduce pore formation in freeze-dried heart valves in a dose-dependent manner, and a 40% sucrose solution was found to be sufficient to diminish pore formation. The presence of pores in freeze-dried valves altered biomechanical characteristics, but did not affect the overall structure and heat stability of the extracellular matrix proteins.

## Results

### Histological evaluation: quantification of tissue porosity

**Figure 1:**
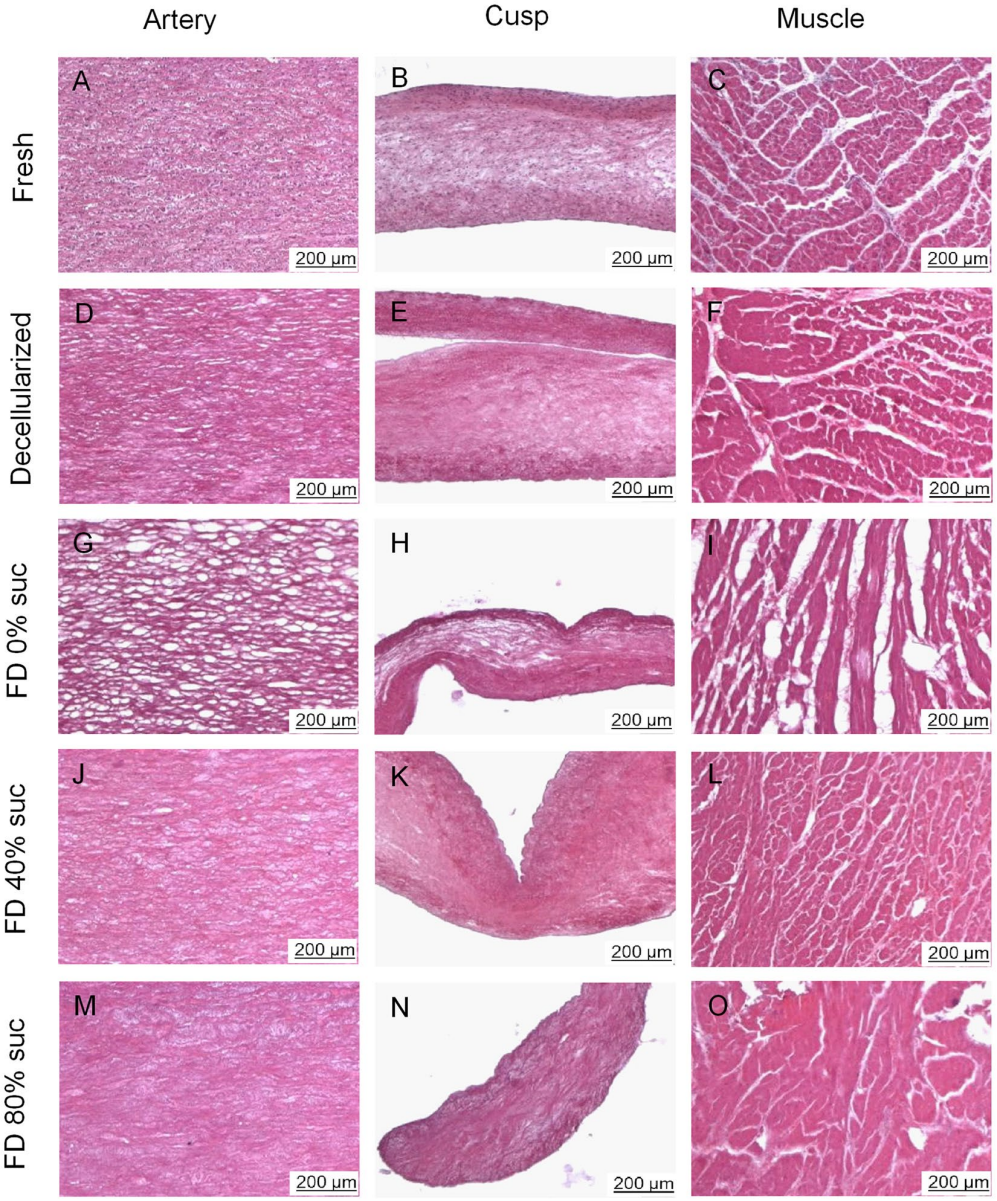
Micrographs of histological sections prepared from pulmonary heart valve tissues, before as well as after decellularization and freeze-drying. Hematoxylin and eosin staining was used to visualize structures in sections of pulmonary artery wall (**A**,**D**,**G**,**J**,**M**), valve cusp (**B**,**E**,**H**,**K**,**N**) and muscle tissue (**C**,**F**,**I**,**L**,**O**). Micrographs are presented of sections from fresh (**A–C**) and decellularized tissue (**D–F**), as well as rehydrated decellularized tissue subjected to freeze drying without protectant (**G–I**), or freeze-dried with 40% sucrose (**J–L**) or 80% sucrose (**M–O**).

The histoarchitecture of decellularized valvular tissues (i.e. pulmonary artery wall, cusp and muscle) was analyzed, both prior to and after freeze-drying (Fig. 1). Cell nuclei, which are visible as dark spots in hematoxylin and eosin-stained specimens of fresh tissue, were found to be absent in decellularized tissue (Fig. 1A–F). The overall ECM structure of decellularized tissue, however, was similar to that of fresh tissue.

**Figure 2:**
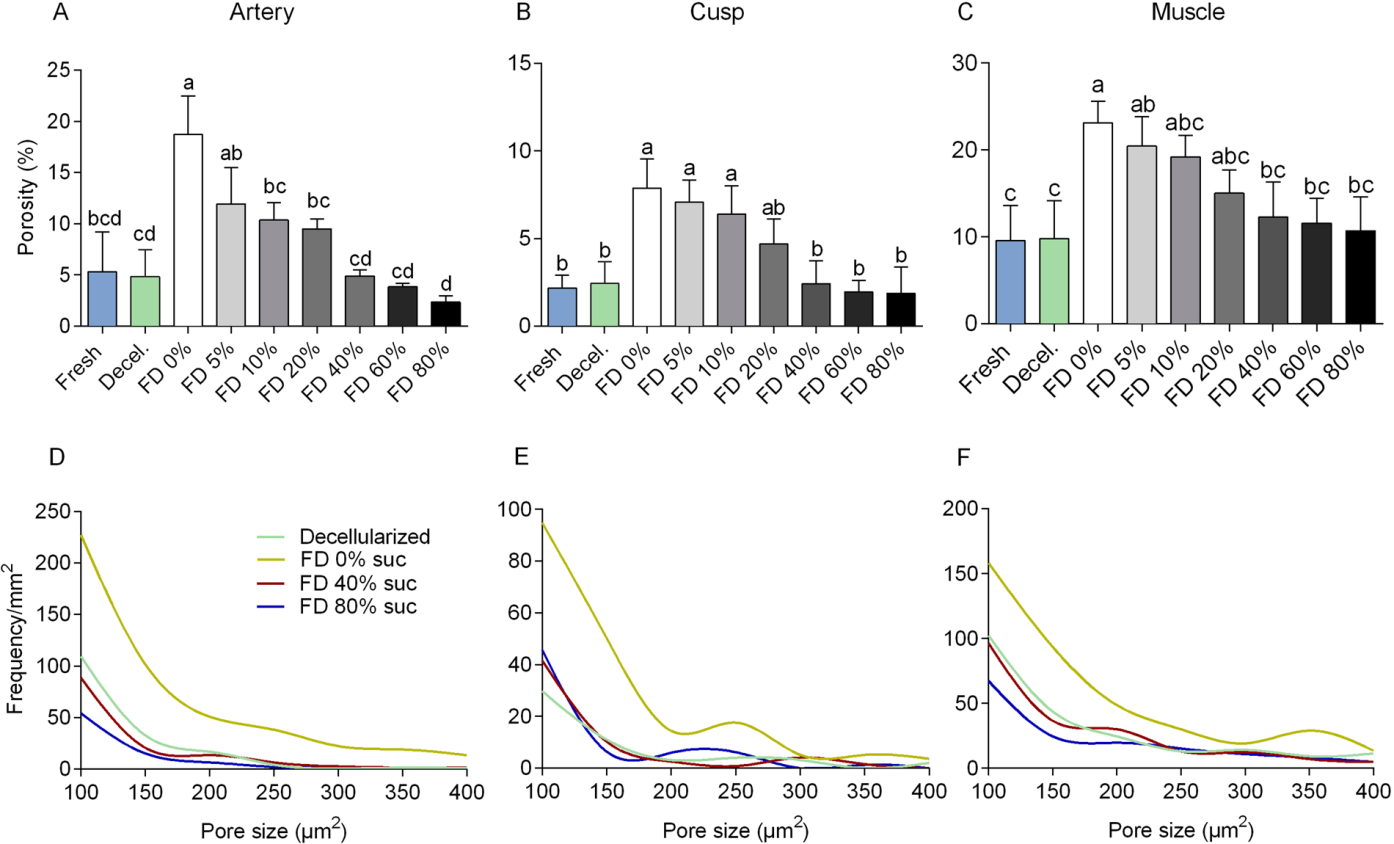
Porosity assessment of pulmonary heart valve tissues, before as well as after decellularization and freeze-drying. Quantification of porosity and pore size distribution via image analysis of micrographs sections of pulmonary artery wall (**A**,**D**), valve cusp (**B**,**E**) and muscle tissue (**C**,**F**). Relative (i.e. percentage) ‘empty’ tissue areas were determined as a measure for porosity (**A–C**). Mean ± standard deviations were calculated from three different specimens, and different letters represent significant differences between groups (p ≤ 0.05). Pore size was measured and its frequency was plotted for decellularized tissue (green line) as well as decellularized tissues freeze-dried without protectants (yellow line) or supplemented with 40% sucrose (red line) or 80% sucrose (blue line).

Valves that were subjected to freeze-drying without protectants exhibited empty areas/pores (Fig. 1G–I). Pores can likely be attributed to ice crystal formation in the scaffolds^22^. The porosity of the various freeze-dried heart valve sections (cusp, artery, muscle), which is evident from the empty spaces in the histoarchitecture, was found to be diminished when sucrose was added as a protective agent in all valve sections (Fig. 1J–O). Tissue porosity of valves freeze-dried with varying sucrose concentration was quantified from the total area of empty spaces in the tissue using image analysis (Fig. 2A–C). Porosity was found to be significantly increased after freeze-drying without protectants for all three valve sections, and it decreased with increasing sucrose concentration in a dose-dependent manner. Porosity of heart valves freeze-dried with 40, 60 or 80% sucrose did not significantly differ from that of valves not exposed to freeze-drying, for all three valve sections. This indicates that treatment with minimally 40% sucrose is required to avoid pore formation in any of the freeze-dried heart valve sections. Histograms of the pore size distribution showed that the increased porosity in non-protected freeze-dried heart valves mostly originates from pores with sizes ranging from 100 to 400 μm^2^ (Fig. 2D–F).

### Ice crystal formation

**Figure 3:**
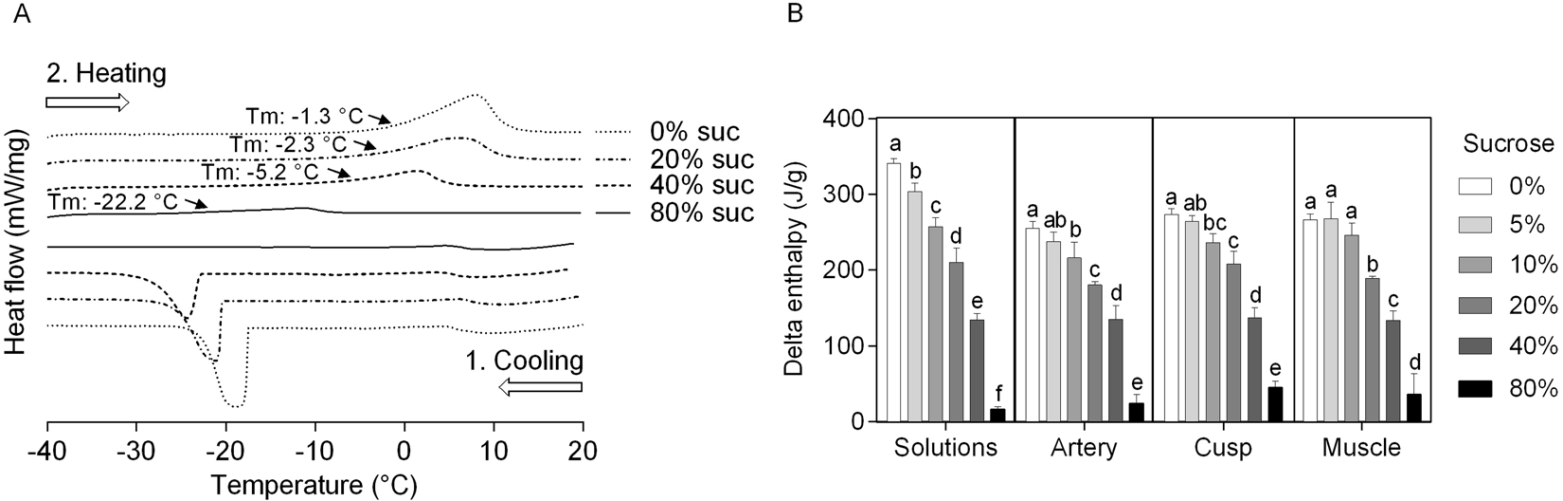
Ice crystal formation in pulmonary heart valve tissues as assessed using DSC. Panel (**A**) shows thermograms of decellularized cusps without protectant or supplemented with sucrose (0%: dotted line, 20%: dashed/dotted line, 40%: dashed line 80%: solid line). Ice formation and melting are evident as exothermic and endothermic peaks during cooling (1), and warming (2), respectively. The specific enthalpy of ice melting (ΔH) was determined from such thermograms (**B**) for artery, cusp and muscle tissue without protectant or supplemented with 0–80% sucrose (grayscale bars). Mean ± standard deviations were calculated from three different specimens, and different letters represent significant differences between groups (p ≤ 0.05).

It is plausible to assume that tissue porosity in freeze-dried tissue is the result of ice crystal formation during freezing that is subsequently removed upon sublimation in the drying step. The amount of ice that was formed during freezing was studied using DSC. Representative DSC thermograms of cusps that were frozen and thawed in the presence of increasing sucrose concentrations are shown in Fig. 3A. Ice formation and melting are visible as exothermic and endothermic peaks, respectively. Enthalpy values, which were derived from the areas under the peaks, reflect the amount of ice that is formed. The ice melting temperature decreased with increasing sucrose concentration, from −1.3 ± 0.3 to −22.2 ± 1.5 °C for 0 and 80% sucrose, respectively. For the cusp tissue the enthalpy of the melting peak decreased with increasing sucrose concentration (ΔH: 273.4 ± 7.5 J g^−1^ for water, and 45.0 ± 8.8 J g^−1^ for 80% sucrose). Similar trends in peak enthalpy values were observed for the other tissue samples, and also for pure sucrose solutions (Fig. 3B). In the presence of 5% sucrose, the enthalpy of the ice melting peak was not significantly different from that in the absence of sucrose, for all three valve tissue sections. In the presence of 40% sucrose, the peak enthalpy was significantly reduced (i.e. 137.2 ± 13.1 J g^−1^ for the cusp sample), but it was still significantly higher than that in the presence of 80% sucrose. Apparently the amount of ice that is formed in the presence of 40% sucrose can be tolerated and does not result in formation of pores.

### Proteins in freeze-dried heart valves

**Figure 4:**
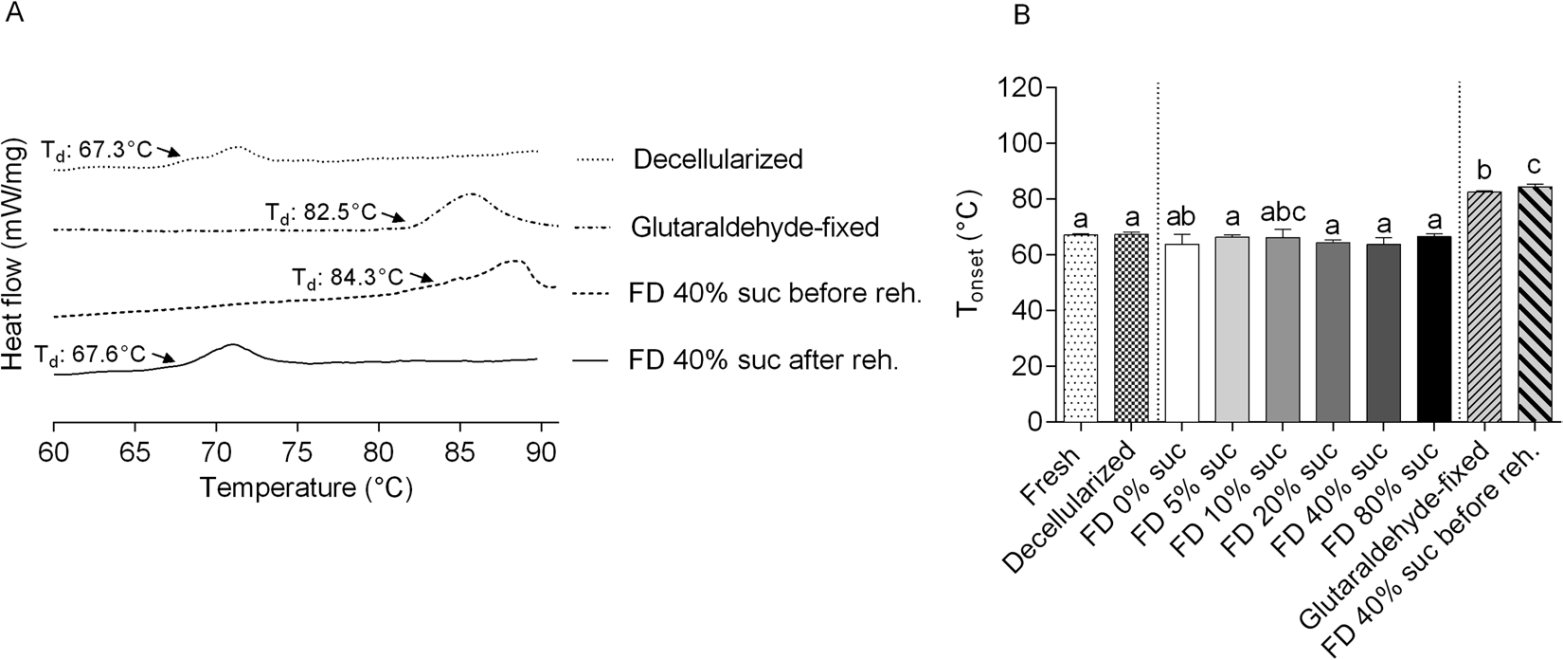
Protein denaturation temperatures in decellularized cusps, determined using DSC. Panel (**A**) shows thermograms of decellularized (dotted line) and glutaraldehyde-fixed (dashed/dotted line) samples. Furthermore, traces are shown for tissue after freeze-drying with 40% sucrose, both in the dry state (dashed) and after rehydration (solid line). The onset denaturation temperature was derived from such thermograms and is presented in panel (**B**). Protein denaturation temperatures were determined for fresh (bar with dots) and decellularized cusps (bar with squares), as well as rehydrated samples exposed to freeze-drying with 0–80% sucrose (grayscale bars). Furthermore, bars are shown for glutaraldehyde-fixed tissue (bars with upward diagonal lines) and freeze-dried tissue in dry state (bars with downward diagonal lines). Mean ± standard deviations were calculated from three different specimens, and different letters represent significant differences between groups (p ≤ 0.05).

DSC was used to determine protein denaturation profiles of the heart valve treatment groups, which provide a comparison based on thermal fingerprints of the samples. Changes in matrix proteins that are induced by processing (i.e. freeze-drying) will alter denaturation characteristics. Protein denaturation is evident as an endothermic event in the DSC thermograms (Fig. 4A). For fresh and decellularized tissues, the onset temperature of protein denaturation, T_onset_, occurs around 66 °C in agreement with previous findings^24^. For comparison, T_onset_ of glutaraldehyde-fixed crosslinked/denatured tissues occurs at a much higher temperature (82.5 ± 0.5 °C)^25^. Freeze-dried tissue (40% sucrose) started to denature at an onset temperature of 84.3 ± 0.9 °C, when the sample was analyzed in the dried state. This increase in denaturation temperature, however, was reversible since after rehydration the DSC thermogram resembled that of non-freeze-dried samples. The onset temperatures of protein denaturation were determined for the different treatment groups with the results shown in Fig. 4B. Decellularized cusps were found to have a T_onset_ of 67.3 ± 0.7 °C. No significant differences in T_onset_ were found among fresh, decellularized and rehydrated tissues that were freeze-dried in the presence of 0–80% sucrose (T_onset_ around 66 °C for all treatment groups).

**Figure 5:**
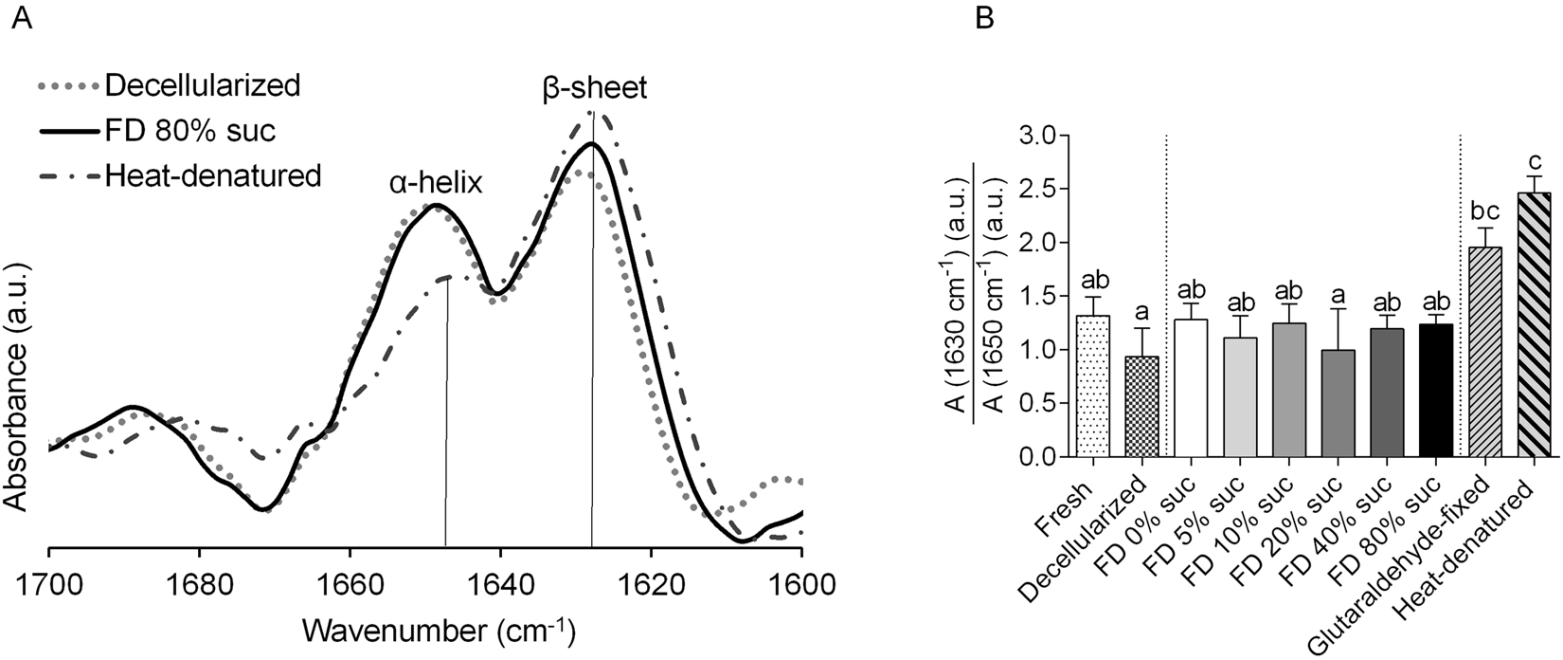
Overall protein secondary structure of decellularized cusps, determined using FTIR. Panel (**A**) shows second derivative spectra in the 1700‒1600 cm^−1^ spectral region, for decellularized (dotted line) and rehydrated tissue exposed to freeze-drying with 40% sucrose (solid line). For comparison, heat-denatured tissue is also shown (dashed/dotted line). Spectra were normalized, and the ratio in α-helical (~1650 cm^−1^) versus β-sheet structures (~1630 cm^−1^) was determined, and presented in panel (**B**). This was done for fresh (bar with dots) and decellularized (bar with squares) cusps as well as rehydrated samples that were freeze-dried with 0–80% sucrose (grayscale bars). Furthermore, bars are shown for glutaraldehyde-fixed samples (bars with upward diagonal lines) and heat-denatured samples (bars with downward diagonal lines). Mean ± standard deviations were calculated from three different specimens, and different letters represent significant differences between groups (p ≤ 0.05).

FTIR was used to evaluate possible changes in the overall protein secondary structure after freeze-drying. Figure 5A shows second derivative spectra of the amide-I region of decellularized cusps prior to freeze-drying and after freeze-drying and rehydration together with a spectrum of heat-denatured tissue. Absorbance bands at 1650 and 1630 cm^−1^ can be assigned to α-helical and β-sheet structures, respectively^26^. Band intensity ratios of α-helical versus β-sheet structures were calculated (Fig. 5B) to reveal differences in protein secondary structure among the treatment groups. Both, glutaraldehyde-fixed and heat-denatured tissue showed a significant increase in the β-sheet/α-helical band ratio, indicating that cross-linking and heat denaturation cause an increase in the relative content of β-sheet structures. The overall protein secondary structure of rehydrated tissues (β-sheet/α-helical band ratio), which were exposed to freeze-drying with 0–80% sucrose, was not significantly different from that of fresh and decellularized tissues (Fig. 5B).

**Figure 6:**
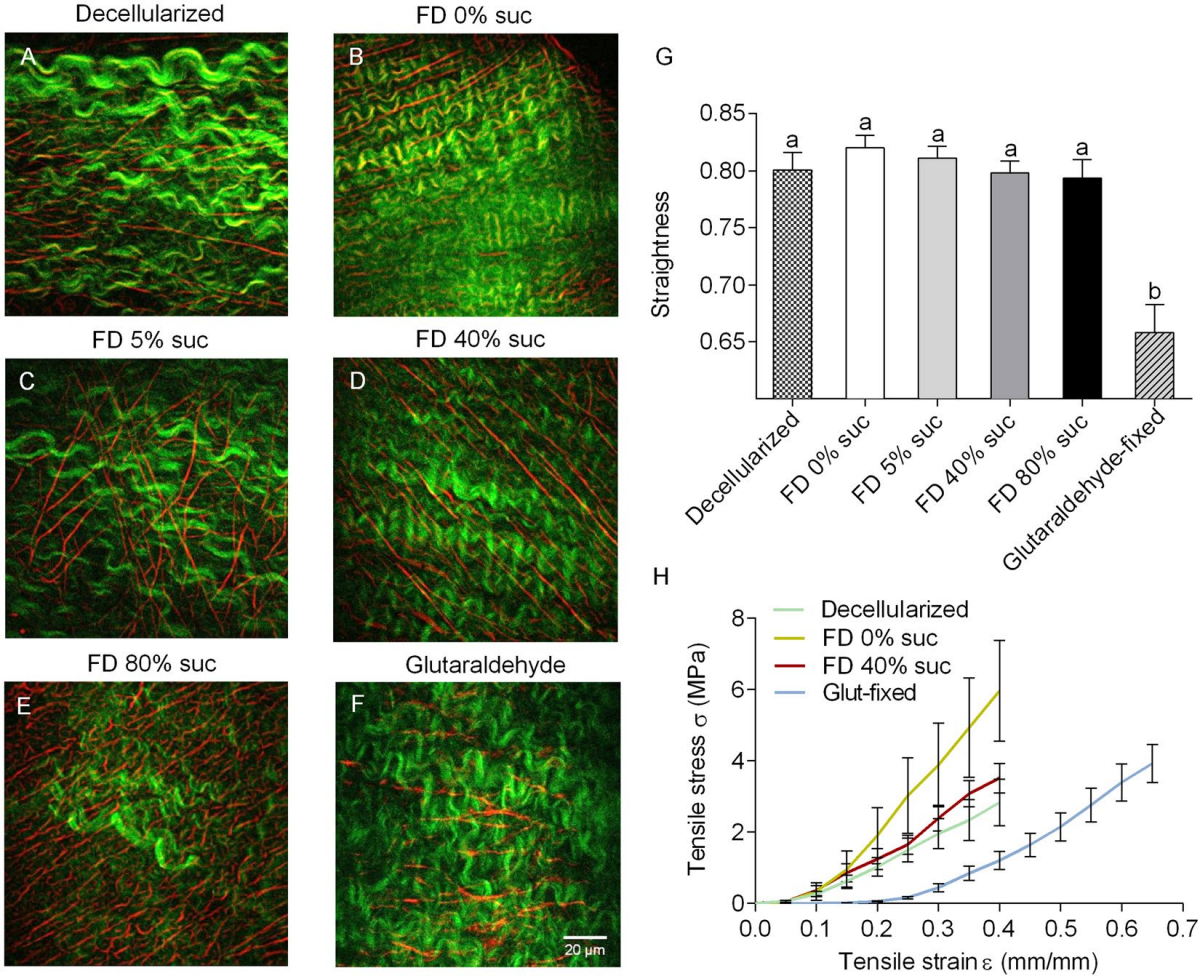
Collagen and elastin microstructure as well as biomechanical properties of freeze-dried pulmonary heart valve cusps. In TPEF/SHG multiphoton images (**A‒F**) the distribution and appearance of collagen bundles (green) and elastic fibers (red) is shown for decellularized cusps (**A**), and rehydrated decellularized cusps subjected to freeze-drying without protectant (**B**) or supplemented with 5% (**C**), 40% (**D**), or 80% sucrose (**E**). Furthermore, an image is shown for a glutaraldehyde-fixed cusp (**F**). In panel (**G**), quantification of collagen waviness is presented, obtained after image analysis. Mean ± standard errors are reported and different letters represent significant differences between groups (p ≤ 0.05). From biomechanical tests, averaged stress-strain curves (n = 6) were calculated (**H**). Curves are shown for decellularized cusps (green line) and rehydrated decellularized cusps which were subjected to freeze-drying without protectants (yellow line) or 40% sucrose (red line). For comparison, a curve is shown for glutaraldehyde-fixed cusps (light blue).

Multiphoton microscopy was used to assess intactness and microstructure of collagen and elastin in freeze-dried valves. For analysis of respectively collagen and elastin, second harmonic generation (SHG) and two-photon excited fluorescence (TPEF) were used. Figure 6A–F shows representative TPEF/SHG images in decellularized cusps, and rehydrated decellularized cusps which were subjected to freeze-drying with 0–80% sucrose. Furthermore, for comparison, a micrograph of glutaraldehyde-fixed cusps is shown. For decellularized tissue not subjected to freeze-drying, collagen (indicated in green) show a fine bundle-like morphology, whereas elastic fibers (indicated in red) are evident as long rope-like structures. Overall, collagen and elastin structures in cusps subjected to freeze-drying and rehydration were found to be similar compared to control cusps not exposed to freeze-drying with multiple layers of long rope elastic fibers and collagen bundles maintaining their degree of fiber orientations. By contrast, collagen bundles in glutaraldehyde-fixed cusps displayed a wavier structure with different fiber orientations (Fig. 6F). Straightness of collagen was significantly affected by glutaraldehyde fixation (0.8 ± 0.015 for the control, and 0.66 ± 0.025 for the glutaraldehyde-fixed cusps) (Fig. 6G). The straightness of rehydrated freeze-dried cusps seemed increased in the absence of protectants and decreased in a dose-dependent manner with increasing sucrose concentrations. These effects, however, were not statistically significant. The estimated size of collagen bundles and the volume density of collagen and elastin measured by pixel intensity did not show significant differences among the tested groups.

### Biomechanical properties of freeze-dried heart valves

**Figure 7:**
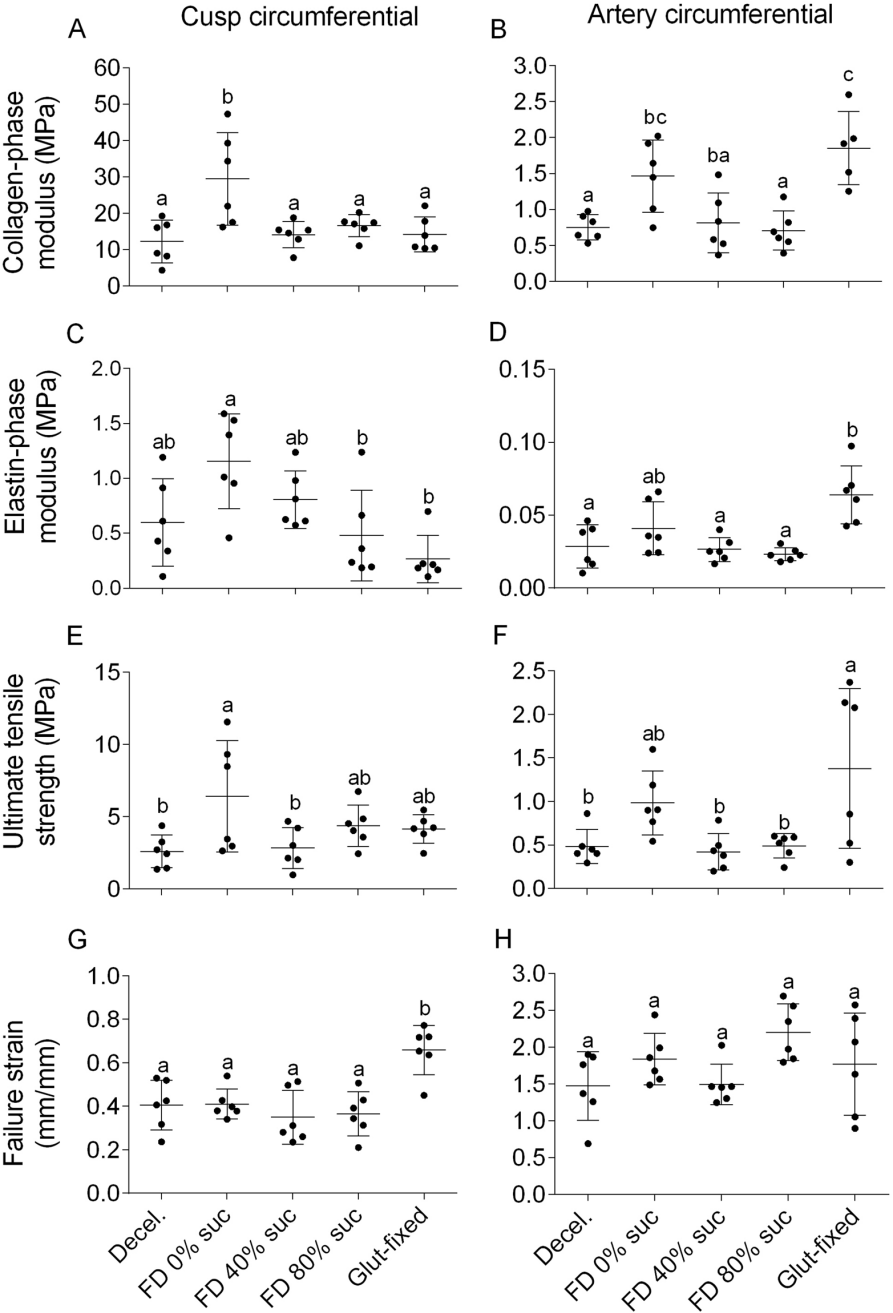
Biomechanical properties of pulmonary decellularized heart valve tissues, both before and after freeze-drying. Freeze-drying was done without protectants as well as 40 or 80% sucrose, and tissues were rehydrated prior to analysis. The E-modulus was calculated from the slope of the collagen and the elastin phase, whereas values for the failure strain (ε_UTS_) and ultimate tensile strength (σ_UTS_) were obtained from the tissue failure point. Failure was taken to occur when the first decrease in load was detected during extension. Tests in circumferential direction are shown using cusp samples (**A**,**C**,**E**,**G**), and artery samples (**B**,**D**,**F**,**H**). E-modulus in the collagen phase (**A,B**), E-modulus in the elastin phase (**C,D**), ultimate tensile strength (**E,F**) and failure strain (**G,H**) values were determined. All acquired values are indicated (points), as well as mean values and standard deviations (lines). Different letters represent significant differences between groups (p ≤ 0.05).

Biomechanical testing was performed on decellularized heart valve artery and cusp sections, both before as well as after freeze-drying and rehydration. Studies with freeze-dried valves were done with three treatment groups: 0, 40, and 80% sucrose. Figure 6H shows stress-strain curves of cusps. The stress-strain curve of cusps that were freeze-dried in the presence of 40% sucrose closely resembles that of control tissue not subjected to freeze-drying, whereas the curve of cusps freeze-dried in the absence of sucrose differs from both these treatment groups. Glutaraldehyde-fixed cusps studied for comparison, show a clearly altered stress-strain curve. Biomechanical testing was done in circumferential as well as radial (or longitudinal) direction for both cusps and artery sections, and characteristic biomechanical parameters were derived from the stress-strain curves. Only data in circumferential direction are shown (Fig. 7), but similar behavior was observed in radial (or longitudinal) direction. Freeze-drying without protectants resulted in a significant increase in the collagen phase moduli of the cusps (p = 0.014) and the artery wall (p = 0.008), and the ultimate tensile strength of cusp (p = 0.043) and artery wall (p = 0.015) compared to samples that were not freeze-dried. The increase in the elastin and collagen phase moduli implied an increased resistance to deformation and decreased tissue compliance. The biomechanical parameters of the tissues that were subjected to freeze-drying with 40 or 80% sucrose were grossly preserved and they were not significantly different from those of decellularized tissues not exposed to freeze-drying. The biomechanical properties of glutaraldehyde-fixed groups were significantly different compared to the control tissue. With regards to the arterial tissue, the modulus of the collagen (p = 0.001) and elastin phases (p = 0.006) increased significantly compared to the decellularized tissue. In contrast with this, the increased failure strain of the glutaraldehyde-fixed cusps indicated that they have become more compliant, suggesting increased levels of deformation for the same levels of applied stress compared to the controls.

## Discussion

Sucrose-protected freeze-dried decellularized pulmonary heart valves were recently successfully used for transplantation in sheep in which their clinical performance was tested^23^. The valves were recovered 6 months after transplantation and found to be repopulated with autologous cells, suggesting freeze-drying does not affect the growth potential of decellularized valves.

In previous studies, we have shown that a 5% sucrose solution is not sufficient to avoid pore formation in freeze-dried heart valves, and that pore formation is diminished using a 80% sucrose solution^21,22^. In the above mentioned animal trial the protocol using 80% sucrose for freeze-drying was tested. For practical applications, and reduction of processing costs, it is of great interest to decrease the amount of sucrose needed for freeze-drying of heart valves. Here we performed a detailed sucrose concentration study and evaluated its effect on pore formation, extracellular matrix proteins and tissue biomechanical parameters.

Porosity depends on the location and kind of tissue, which was carefully considered here in order to perform a comparative study on pore formation caused by freeze-drying in the different heart valve tissue sections. Pretreatment with sucrose reduced pore formation in a dose-dependent manner, and a 40% sucrose solution prior to freeze-drying was found to be sufficient to completely diminish pore formation. Ice crystal formation is still clearly measurable in scaffolds pre-treated with 40% sucrose, but apparently the amount of ice that is formed after such incubation can be tolerated and does not result in ice crystal damage and formation of pores. The overall scaffold histoarchitecture of valves freeze-dried with 80% sucrose was found to be similar to that of valves freeze-dried with 40% sucrose. A tendency can be observed that the percentage of empty spaces further decreases at concentrations greater than 40%, which can be particularly seen in the artery section, but this effect was not significant. Exposure to high-concentration sucrose solutions results in compaction of the tissue, because the tissue will dehydrate upon immersion in the sucrose solution due to the osmotic gradient that is being created. This in turn will result in less ice formation and less pore formation upon freeze-drying. In addition to the well-known lyoprotective effects of sucrose such as water replacement^27^ and glass formation at room temperature^28^, tissue dehydration upon exposure to high-concentration sucrose solutions prior to freeze-drying likely also plays an important role in tissue protection during freeze-drying.

Haugh *et al*.^29^ reported that ice crystals and pore size can be controlled by modifying the final freezing temperature in collagen-glycosaminoglycan scaffolds. It was found that modifications of the final freezing temperature (−10 °C to −70 °C) can be used to produce a wide range of pore sizes (325–85 μm). Lowering the operating temperature for freeze-drying, however, would result in a drastic increase in the total operating time needed for complete drying^30–32^. We note here that it is predominantly the freeze-drying formulation rather than the freeze-drying parameters that determine the characteristics of freeze-dried valves.

Protein stability and the overall protein secondary structure were well preserved in rehydrated tissues after freeze-drying, irrespective of the presence or absence of sucrose. As expected, the protein denaturation temperature of freeze-dried tissue is increased when the tissue is analyzed in the dried state. The protein denaturation temperature of freeze-dried proteins is determined by the residual water content and by excipients^33^. Sucrose decreases the protein denaturation temperature of dried proteins^34^, but at the same time sucrose stabilizes proteins during drying by preventing dehydration-induced conformational changes and by forming a protective glassy state^35,36^. The increase in the protein denaturation temperature of freeze-dried tissue is reversible, since after rehydration the protein denaturation temperature is similar to that of tissue not subjected to freeze-drying, indicating that proteins are little affected by the freeze-drying process. The FTIR and multiphoton imaging studies corroborate these findings and show that the overall structure of the matrix proteins is not affected by the freeze-drying process. Collagen is relatively stable during freezing and drying. Beghé *et al*.^37^ showed that collagen can be lyophilized without protectants while maintaining its properties and structure.

Multiphoton imaging showed that the architecture of rehydrated freeze-dried cusps is well preserved with multiple layers of collagen bundles comprised of some apparent symmetrical orientations irrespective of the sucrose concentration used as lyoprotectant. By contrast, glutaraldehyde-fixed tissue (resembling the structure in bioprosthethic valves) displayed a wavy structure comprised of elastic fibers and a different space arrangement of collagen bundles. It has been reported that both SHG and TPEP can detect glutaraldehyde-induced alterations in the microscopic structure and intermolecular crosslinks of collagen^38^. Alterations in biomechanical properties of cusps could be correlated with the straightness of collagen bundles. Fibers that do not bear load are assumed to be in a wavy state, whereas bundles are elongated when subjected to a certain strain^39^. Apparently freeze drying without protectants loosens the collagen bundles resulting in steeper stress-strain curve, whereas glutaraldehyde fixation makes the collagen bundles wavier resulting in a less steep stress-strain curve and drastically increases the failure strain in cusps. Moreover, freeze-drying without protectants increases the collagen modulus of cusps and artery walls making the tissues stiffer.

Proper heart valve functioning depends on the biomechanical properties of cusps^40–42^. They have to be flexible, resistant and provide an adequate rigid structural strength. A low E-modulus results in a better hydrodynamic function, allowing easy opening and closing of the cusps, whereas a high E-modulus is better for cusp durability^43^. The same principles may be applied to the artery tissues. In this case, the macroscopic collagen alignment in the cusps (collagen cords) transfers forces from the cusps to the artery wall, which should have an adequate strength, flexibility, and durability^44^.

Cusp deformation during the cardiac cycle is facilitated by biomechanical interactions between collagen an elastin where the E-modulus plays an important role. During every cardiac cycle heart valve leaflets are exposed to complex mechanical stress, including: sheer stress due to blood flow (open valve), flexure (opening and closing), and tension (closed valve)^45^. When the valve is open, elastin in extracellular matrix stretches, and collagen crimps and corrugates. When the valve is closed, collagen is unfolded and the force is shifted from elastin to collagen^44^. It is shown that the E-modulus values in both the collagen and the elastin phase are well preserved when tissues were freeze-dried with 40 or 80% sucrose. Freeze-drying without protectants, however, resulted in alterations of the E-modulus values both in artery as well as cusp tissues, in both directions. The greater E-modulus and ultimate tensile strength observed in the freeze-dried tissues without protectant can likely be attributed to the effects of pores on the strength of the tissue. The spherical pores formed by freeze-drying may better resist mechanical loading, therefore reinforcing the collagen in the scaffolds^19^. A too high E-modulus, which is also observed in the glutaraldehyde-fixed artery, could result in valve malfunctioning. Glutaraldehyde fixation adversely increases rigidity or stiffness affecting the tissue-bending properties which may lead to tissue buckling when the tissue flexes during the cardiac cycle, producing points of stress concentration and tearing^46^. It also causes damage in the membrane leading to calcium phosphate crystal nucleation, and valve failure due to stenosis^47^. In the artery we found that glutaraldehyde fixation caused an increase in the elastin and collagen phase moduli, which implies an increased resistance to deformation and decreased tissue compliance. Glutaraldehyde-fixed cusps however, showed a much larger elastin phase than the other groups, depicting alterations in the elastin and collagen network.

In conclusion, without protective measures, freeze-drying heart valves results in matrices that have pores and altered biomechanical properties. Pore formation changes biomechanical characteristics of freeze-dried heart valves, whereas tissue proteins are not affected by pore formation in terms of overall protein secondary structure and protein quaternary structure. By freeze-drying using a sucrose solution at sufficiently high concentrations therewith reducing the amount of ice that is formed during freezing, porosity can be diminished and biomechanical properties resemble those of valves not exposed to freeze-drying. Exposure to a 40% sucrose solution prior to freeze-drying appears to be sufficient to avoid pore formation in freeze-dried heart valves and to preserve the collagen and elastin structure as well as biomechanical characteristics.

## Methods

### Heart valve decellularization

Fresh porcine hearts were obtained from a local abattoir and transported to the lab in phosphate buffered saline (PBS; 137 mM NaCl, 27 mM KCl, 10 mM Na_2_HPO_4_, 1.8 mM KH_2_PO_4_, pH 7.4) supplemented with 1% (v/v) penicillin. Pulmonary valves were excised from the hearts. After removing excess fat and connective tissue the valves were washed for 5 min in 7.5% (w/v) iodine solution and PBS. Valves were decellularized as previously described^48^. Briefly, valves were treated with 0.5% (v/v) Triton X-100 for 24 h under orbital shaking, followed by 24 h treatment with 0.5% (w/v) SDS, and 24 h washing with deionized water. The detergent solutions were replaced by fresh solutions every 12 h. Decellularization was completed by two 12 h washing cycles in distilled water, followed by 12 washing cycles of 12 h each in PBS. Valves were stored in PBS at 4 °C and used within one week.

### Freeze-drying

Decellularized pulmonary valves were cut along their longitudinal direction, between the cusps, resulting in three pieces. These were assigned to the following experimental groups: decellularized tissue, decellularized tissue freeze-dried without protectants, as well as freeze-dried with 5, 10, 20, 40, 60 or 80% (w/v) sucrose. For freeze-drying without protectants, decellularized tissue was maintained in PBS. Preloading with sucrose and freeze-drying were done as previously described^22^. Decellularized valves were first incubated in 5% sucrose solution, for 4 h at 37 °C. Then, valves were transferred to 50 mL of the respective sucrose solution in a flask, and incubated for 30 min while changing the solution with fresh solution every 10 min. Immersing the valves in high sucrose concentrations results in transport of water from the tissue to the solution (osmotic gradient), and a further increase of the sucrose concentration in the tissue. Incubations were done on an orbital shaker. Incubation times were estimated based on previous measurements on sucrose diffusion into the various heart valve tissue parts^22^.

The valves were transferred to petri dishes and placed in a freeze-dryer with temperature controlled shelves (Virtis Advantage Plus Benchtop freeze dryer; SP scientific, Warminster, PA). Samples were subjected to slow freezing from room temperature down to −30 °C at 1 °C min^−1^. Primary drying was performed for 7 h, at **−**30 °C and 60 mTorr. For secondary drying, the shelf temperature was slowly increased to 40 °C at 0.1 °C min^−1^ and samples were maintained at 40 °C and 60 mTorr for 30 min, after which the shelf temperature was reduced to 20 °C and kept at 20 °C until samples were taken out. Dried valves were stored in sealed petri dishes at 4 °C, until use within one week. The valves were rehydrated by adding water, which was followed by washing in PBS (3 × 30 min, at 37 °C) to remove sucrose.

### Histological evaluation and porosity

Microscopy was used for histological evaluation of (freeze-dried) decellularized heart valve tissues. Tissue was fixed, embedded in paraffin, sectioned and stained with hematoxylin and eosin as described in detail elsewhere^21^. Micrographs were obtained with a light microscope (BX40; Olympus, Tokyo, Japan) equipped with camera and accompanying photo documentation software (Axiovision; Zeiss, Hamburg, Germany). Similar illumination, exposure, contrast and background settings were used for all samples. Total porosity and pore size distribution was studied on tissues after decellularization and/or freeze-drying on three different heart valves per group. To consider a tridimensional approach, three microscopic fields per sample corresponding to 0.609 mm^2^ were analyzed at different depth of the tissue and studied using ImageJ software^49^. For the artery wall, porosity was studied in the tunica media layer of the tubular part. In the cups porosity was measured in the central part including the three different layers (fibrosa, spongiosa and ventricularis), whereas for the muscle section, porosity was assessed in the extracellular matrix of smooth muscle cells. In short, brightness/contrast level of micrographs (100× magnification) were adjusted and converted to 8-bit grayscale images. Lower and upper threshold levels were set at respectively 0, and 170-225 for all images. Then, the relative area (i.e. percentage) within a tissue-section which was not covered with tissue (i.e. pores/empty space) was measured. A scale bar of a known distance was used for size calibration, and the pore size frequency was determined.

### Differential scanning calorimetry

Differential scanning calorimetry (DSC) measurements were done using a Netzsch DSC 204F1 Phoenix instrument (Netzsch Geraetebau GmbH, Selb, Germany), according to the instructions provided by the manufacturer. Small pieces with a 5 mm diameter were cut from artery, cusp and muscle tissue, using a biopsy punch (Stiefel, Wächtersbach, Germany). Weights were determined and samples were transferred into 25-µL aluminum DSC pans, which were hermetically sealed. An empty pan was used as a reference. To study ice crystal formation and melting, specimens infiltrated with sucrose were cooled from 20 °C down to −40 °C, followed by heating to 20 °C, at 10 °C min^−1^, while monitoring the heat flow. Protein denaturation profiles of fresh, decellularized, and (rehydrated) freeze-dried tissues were measured by heating samples from 20 °C to 100 °C at 10 °C min^−1^. DSC traces were analyzed using Proteus thermal analysis software (Netzsch Geraetebau GmbH) as previously described^24^.

### Fourier transform infrared spectroscopy

Infrared absorption spectra were recorded using a Fourier transform infrared spectrometer (FTIR) (Perkin Elmer, Waltham, MA, CT, USA), equipped with a triglycine sulfate detector and an attenuated total reflection (ATR) accessory with pressure arm and diamond/ZnSe crystal. The acquisition parameters were: 4 cm^−1^ resolution, 8 co-added interferograms, 4000–900 cm^−1^ wavenumber range. An automatic CO_2_/H_2_O vapor correction algorithm was used during recording of the spectra. Spectra analysis was done using PerkinElmer software (Perkin Elmer) and Omnic software (Thermo Nicolet, Madison, WI, USA). Tissue pieces with a 5 mm diameter were cut using a biopsy punch as described above. Prior to FTIR analyses, samples were incubated (2 × 30 min) in D_2_O (Sigma-Aldrich Chemie GmbH, Munich, Germany) in order to avoid interference from H_2_O absorbance bands in the protein region of the spectrum. The ATR pressure arm was used to mount the samples on the measurement surface area. Similar pressure conditions were used for all samples tested. To evaluate the overall protein secondary structure, the amide-I band region (1600–1700 cm^−1^) was selected. Second derivative spectra with a 13-point smoothing factor were calculated to resolve contributions from α-helical structures (~1650 cm^−1^) and β-sheet structures (~1630 cm^−1^) more clearly.

For comparison, fixed tissue and denatured tissue were also analyzed. Fixation was done via exposure to 0.6% glutaraldehyde for 24 h at room temperature, and thermal denaturation was achieved by 30 min incubation of hydrated tissue in a 90 °C water bath.

### Multiphoton imaging

Multiphoton microscopy was used to observe extracellular matrix components in the tissues. The spectral imaging system used includes a CellSurgeon’ scanning inverted microscope (Axio Observer; Zeiss, Jena, Germany) from LLS ROWIAK (Hannover, Germany) equipped with a femtosecond mode-locked near-infrared 750–1050 nm titanium-sapphire laser (Coherent Chameleon; Santa Clara, USA). For high-resolution imaging, an oil immersion objective (Apochromat, magnification: 40×, numerical aperture: 1.3) was used. The collagen microstructure was visualized using second harmonic generation (SHG) signals with an excitation wavelength set at 880 nm and emission at 440 nm, whereas morphology of elastin was visualized using two-photon excited fluorescence (TPEF) signals with excitation at 780 nm and emission at 390 nm. A Hamamatsu H7422-20 photomultiplier was used to collect the SHG and TPEF signals, and a Croma HQ 460/80m-2p filter was used in both cases. ImageJ (‘tubeness’ filter) was used to increase the contrast of the TPEF images, and collagen and elastin were color-coded in green and red after which they were merged. Waviness of collagen was analyzed in 17 collagen bundles per group as previously described by Rezakhaniha *et al*.^39^. Using the ImageJ plug-in ‘NeuronJ’ the straightness parameter Ps was calculated:

1 $$Ps={L}_{0}/{L}_{f}$$

where *L*_0_ *is* the distance between visible endpoints of a collagen bundle and *L*_*f*_ the length of the fiber bundle (*L*_*f*_). In addition, the thickness (*t*) was measured in three different locations along each bundle to estimate the size of the bundles ($\widetilde{S}$):

2 $$\tilde{S}={L}_{f}\times \bar{t}$$

### Biomechanical testing

Biomechanical testing was performed using a uniaxial tensile tester (Instron 3365; Instron, Bucks, UK), under low-strain-rate uniaxial tensile loading to failure conditions^50^. The biomechanical properties of decellularized pulmonary artery and cusp tissue, as well as decellularized tissues that were subjected to freeze drying and rehydration (freeze-dried without protectant and with 40 or 80% sucrose). Samples were cut with a bespoke cutter along the circumferential and longitudinal directions in the case of the pulmonary artery, or along the radial direction for the case of the cusps, to obtain specimens with a width of 2.5 mm and a testing length of 5 mm, owing to the limited size of the pulmonary cusps, only 3 radial cusps samples were isolated and tested. Six samples were tested for all other tissue groups. Prior to testing, sample thickness was measured at three point along their long axis, using a thickness gauge (Mitutoyo Europe GmbH, Neuss, Germany; precision: ± 0.01 mm), and their average thickness was recorded. Subsequently, samples were mounted onto a bespoke stainless steel holder that set the gauge thickness of the samples to 5 mm, before the holder was mounted to the tensile tester. Biomechanical testing was conducted in PBS, at 37 °C. Prior to loading**-**to**-**failure, the samples were preconditioned by 50 loading/unloading cycles, up to the transition strain, at a rate of 20 mm min^−1^. Following preconditioning, samples were sequentially stretched-to-failure (at 20 mm min^−1^). The resulting stress-strain curves were used to calculate the elastin and collagen phase moduli (in MPa), together with the failure strain and ultimate tensile strength (in MPa) at the failure point, which was taken as the point at which the first decrease in load during extension was observed.

### Statistical analysis and experimental design

Biomechanical studies were performed using material from six different pulmonary heart valves for each treatment group. Tissue sections were prepared from three different pulmonary heart valves for each group and used to quantify the porosity of the tissue. Moreover, DSC and FTIR studies on ice crystal formation, protein denaturation and secondary structure were performed in triplicates. Statistical significant differences among the test groups were analyzed by one-way analysis of variance (ANOVA) followed by Tukey’s multiple comparisons test. The results were presented as mean ± standard deviation, unless otherwise noted and differences were considered to be statistically significant if p ≤ 0.05.
